# Developmental Morphokinetics and the Transcriptomic Profile of Bovine First-Cleaved Embryos: Normal vs. Abnormal Divisions

**DOI:** 10.3390/ijms27114885

**Published:** 2026-05-28

**Authors:** Ariel Michaelov, Dorit Kalo, Moran Gershoni, Zvi Roth

**Affiliations:** 1 Department of Animal Sciences, Robert H. Smith Faculty of Agriculture, Food and Environment, The Hebrew University, Rehovot 7610001, Israel; 2 Department of Ruminant Science, Institute of Animal Sciences, Agricultural Research Organization, Volcani Institute, Rishon LeZion 7505101, Israel

**Keywords:** abnormal cleavages, bovine, embryo morphokinetic, transcriptome

## Abstract

While early embryonic loss affects the conception rate in lactating cows, the underlying mechanisms remain unknown. Here, we examined whether the developmental morphokinetics and transcriptomic profiles of cleaved embryos are associated with developmental competence. Developing bovine embryos were produced in vitro, and their morphokinetics were monitored through 190 h using a time-lapse system. The proportion of embryos that developed to the blastocyst stage was lower following abnormal cleavage, i.e., reverse, direct, or unequal, relative to normally cleaved embryos (*p* < 0.05). In a second set of experiments, exploratory RNA-seq analysis was performed on first-cleaved embryos, which were individually collected immediately after the first division; embryos were defined by the time-lapse system as normally, directly, or unequally cleaved (*n* = 6 per group), or reverse-cleaved (*n* = 5). Analysis revealed 672 genes that were differentially expressed between normally and abnormally cleaved embryos (adjusted *p* < 0.05). Abnormally cleaved embryos differed in pathways associated with energy production and metabolism. The most profound difference in gene expression (*n* = 632) was between unequally and normally cleaved embryos, mainly in genes affiliated with the oxidative phosphorylation pathway. These findings support the concept that the morphokinetic pattern of early embryonic cleavage is associated with developmental competence. We therefore suggest that the observed differential expression profiles in abnormally cleaved embryos might be involved in the mechanism underlying early embryonic loss.

## 1. Introduction

Embryonic loss has been suggested to reduce fertility in lactating cows [1]. This adverse reproductive event can occur due to several failures, including asynchrony between the embryo and uterine environment [2,3], deficient maternal recognition of pregnancy [4,5,6], endometrial inflammation, uterine disease, or both [7], and hormonal insufficiency [8]. Embryonic death can occur at any stage of development, from the day of fertilization, through early embryonic stages, i.e., from day 7 to 24 post-fertilization, to late embryonic and fetal mortality from day 24 onwards [9]. Nevertheless, most embryonic losses occur between fertilization and maternal pregnancy recognition [2]. A recent meta-analysis in beef cattle, which applied more than 56,000 diagnostics, reported that most embryonic developmental failures occur within the first 7 days post-fertilization and among these, most occur before day 4 post-fertilization. This accounts for 47.9% pregnancy loss from fertilization to day 28 of pregnancy, with 28.4% occurring in the first week [10]. To date, despite its deleterious effects on the cattle industry, the specific causes of early embryonic loss remain unclear. Moreover, there is a lack of tools for studying embryonic development in vivo during the first days post-insemination. Nevertheless, in vitro models in which oocytes are matured, fertilized, and developed into blastocysts in culture allow the study of possible mechanisms that underlie early embryonic loss, such as alterations in developmental morphokinetics or in the transcriptomic profile of the embryo.

Developmental morphokinetics can be efficiently monitored in the embryo by using a state-of-the-art incubator equipped with a time-lapse system. This system enables recording the timing and duration of cell kinetic events and embryo morphology and identifying division patterns. These data enable the prediction of the embryo’s competence to develop to the blastocyst stage [11,12,13,14] as well as its implantation potential [15]. Among these morphokinetic parameters, the timing of the first division is associated with blastocyst formation rates, as early-cleaved embryos are more likely to develop to the blastocyst stage compared with those that divide more slowly [16,17]. Another important factor is the division pattern, as normally cleaved embryos were found to have a higher likelihood of developing to the blastocyst stage [18,19,20]. Abnormally cleaved embryos are characterized by direct, unequal, or reverse divisions. In directly cleaved embryos, a zygote cleaves directly into three or four unevenly sized blastomeres, characterized by a slow growth rate and a reduced proportion of formed blastocysts [18,19]. In unequally cleaved embryos, a zygote cleaves into two unequally sized blastomeres, whereas in reverse-cleaved embryos, the two blastomeres fuse or fail to separate, resulting in a one-cell embryo.

Given the lower ability of abnormally cleaved embryos to develop to the blastocyst stage, it is reasonable to assume that abnormal divisions may underlie, at least in part, early embryonic loss. In support of this assumption, previous studies in bovines found a relatively high frequency of abnormally cleaved embryos, with reported values ranging between 30% and 50% [18,19,20,21]. Yet, despite this high incidence, the mechanism underlying abnormal cleavage remains unclear. Recent studies from our group and others reported that blastocysts developed from normally cleaved embryos differ in their transcriptomic [18] and metabolomic profiles [20] from abnormally cleaved embryos, specifically following direct division. However, these studies mainly focused on blastocysts, i.e., after activation of the embryonic genome, rather than on the first-cleaved embryos, i.e., maternal genome.

The maternal genome controls and supports early embryo development until embryonic genome activation, which in bovine occurs at the 8- to 16-cell stage [22,23]. Proteins and maternal mRNA in the oocyte govern the first mitotic divisions of the preimplantation embryo [24]. To become fully competent, the oocyte undergoes nuclear maturation, which mainly involves chromosomal segregation, and cytoplasmic maturation, which involves organelle reorganization and cytoskeleton dynamics [25]. It also undergoes molecular maturation, which involves transcription, processing, and storage of mRNA and proteins that are subsequently involved in fertilization, pronucleus formation, and the first embryonic division. The follicular cells surrounding the oocyte also contribute to oocyte quality [26]. The gap junctions between the cumulus cells and the oocyte enable the bilateral transfer required for oocyte development and competence [27]. Together, it is reasonable to assume that the oocyte plays a requisite role in the first embryonic division. Moreover, given the lateness of embryonic genome activation, the bovine model is a suitable and powerful tool for studying whether maternal transcripts are involved in the mechanisms underlying the first cleavage.

In the current study, we performed a comprehensive transcriptome analysis to explore the molecular mechanisms that underlie the division patterns of first-cleaved bovine embryos. We evaluated the transcriptomic profile of the first-cleaved embryos, before genome activation. Embryos were therefore collected immediately after the first cleavage pattern was recognized, to minimize transcriptome variability. The cleaved embryos were categorized as normally or abnormally cleaved and then underwent RNA-seq analysis.

## 2. Results

### 2.1. Association Between the First Cleavage Pattern and Further Embryonic Development

To understand the association between the first cleavage pattern and further embryonic development, putative zygotes were cultured in vitro for ~190 h, i.e., until the blastocyst stage. First cleavage patterns were defined as either normal or abnormal (i.e., direct, unequal, or reverse cleavage) retrospectively. Results showed a cleavage rate of 84.8 ± 3.4% and blastocyst formation rate of 20.6 ± 4.1%. There was no difference in the proportion of normally vs. abnormally cleaved embryos (45.6 vs. 41.8%, respectively). However, a higher proportion of normally cleaved embryos developed into blastocysts, relative to the abnormally cleaved (39.6 vs. 8.9%, respectively; *p* < 0.0001). Further analysis revealed that the proportions of cleaved embryos either out of total cumulus oocyte complexes (COCs) or out of total cleaved embryos were higher in the normal cleaved embryos compared with the directly, unequally, and reverse cleaved embryos (*p* < 0.0001; Table 1).

Among the abnormally cleaved embryos (*n* = 123), the proportion of direct cleavage was the highest, followed by the unequal pattern and finally, the reverse pattern (Table 1). No significant difference was found between directly and unequally cleaved embryos in the proportion of embryos that further developed to the blastocyst stage (Table 1).

In addition, the timing of the first cleavage differed between groups; normally cleaved embryos cleaved earlier than directly or unequally cleaved embryos (*p* = 0.001 and *p* = 0.0104, respectively; Table 1). Conversely, the timing of the first division did not differ among the abnormally cleaved embryos.

### 2.2. Association Between the First Cleavage Pattern and Transcriptomic Profile

**Figure 1:**
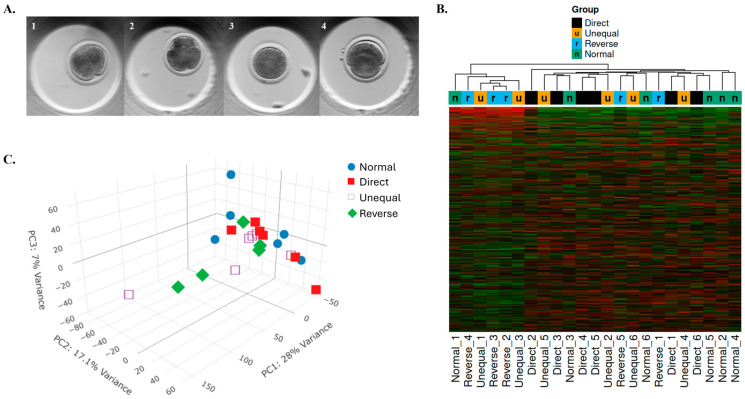
Transcriptome analysis of RNA-seq data from first-cleavage bovine embryos. (**A**) Representative images of first cleaved embryos demonstrating normal (1), direct (2), reverse (3), and unequal (4) cleavage patterns as recorded by the time-lapse system. (**B**) Heatmap of DEGs between abnormal and normal first-cleavage embryos, used to visualize global expression structure and sample-level heterogeneity. Colors indicate the expression levels of annotated DEGs, where red denotes higher expression while green indicates lower expression. Each horizontal line represents a single gene; each column represents a single cleaved embryo sample. (**C**) Principal component analysis (PCA) of the transcriptomic data analyzed by the iDEP browser.

**Figure 2:**
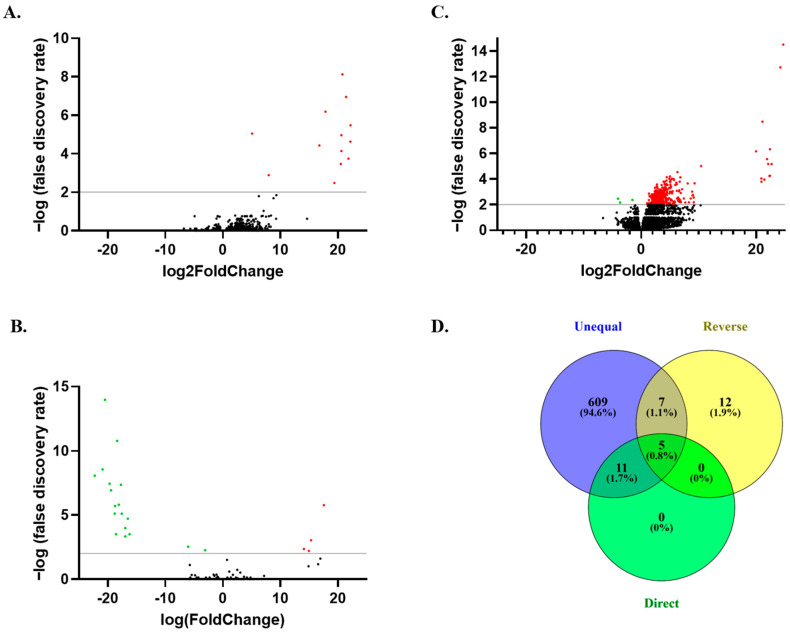
Transcriptomic comparisons between normally cleaved embryos and directly, reversely, or unequally cleaved embryos. (**A**–**C**) Volcano plots showing the log_2_ fold change as a function of the −log_10_ adjusted *p*-value for each abnormal cleavage pattern relative to normal embryos ((**A**), directly cleaved; (**B**), reversely cleaved; (**C**), unequally cleaved embryos). Each dot represents a gene. Significantly up- or down-regulated genes (>2-fold) are highlighted in red or green, respectively. Black dots represent non-significant differential expression. (**D**) Venn diagram showing overlap of DEGs among directly (green, *n* = 16), reverse (yellow, *n* = 24), and unequally (purple, *n* = 632) cleaved embryos.

Having determined the association between cleavage pattern and blastocyst development rate, we proceeded to study the molecular basis of this association by conducting transcriptomic analyses. Differential expression analysis of normally vs. abnormally cleaved embryos revealed 672 differentially expressed genes (DEGs) (adjusted *p* < 0.05), with transcriptomic differences between normally cleaved embryos and each one of the three abnormal cleavage patterns (Figure 1). ANOVA analysis revealed significant differences in gene expression between the experimental groups (*p* < 0.0001). Post hoc Tukey’s HSD tests indicated that embryos with both reverse and unequal cleavage patterns differed significantly from normal embryos (*p* < 0.0001 and *p* < 0.0001, respectively), whereas the directly cleaved pattern did not (*p* = 0.14). Hierarchical clustering of the DEG heatmap (Figure 1B) did not show complete segregation of embryos according to morphokinetic group, indicating substantial embryo-to-embryo variability in the global transcriptomic profiles. This pattern was also reflected in the principal component analysis (PCA) (Figure 1C), where the groups did not form fully separated clusters. Nonetheless, abnormally cleaved embryos displayed broader dispersion across the principal components (PCs), suggesting that abnormal cleavage is associated with increased transcriptomic heterogeneity. In particular, the unequally cleaved samples displayed a distinct pattern of variation, being more dispersed along PC1 while relatively closer along PC2 and PC3. We next performed pairwise differential expression analyses comparing normally cleaved embryos with each abnormal cleavage pattern separately (Figure 2 and Table 2).

### 2.3. Normally vs. Directly Cleaved Embryos

Comparison of transcription patterns between normally and directly cleaved embryos revealed 16 genes significantly differentially expressed between the groups (Table S1), all of which were more highly expressed in the directly cleaved embryos (Figure 2A). 

Significantly DEG-enriched pathways in directly cleaved embryos that are associated with oocyte quality or early embryonic development, including the steroid hormone biogenesis pathway, are shown in Table 2.

### 2.4. Normally vs. Reverse-Cleaved Embryos

Comparing the transcriptomes of normally vs. reverse-cleaved embryos (Figure 2B) revealed 24 DEGs (Table S2), of which 19 were downregulated in the reversely cleaved embryos. The five genes found to be upregulated in the reverse-cleaved embryos are melanoma-associated antigen H1 (*MAGEH1*), IFN-induced transmembrane protein 1 (*IFITM1*), IFN-stimulated gene 15 (*ISG15*), junctional adhesion molecule 2 (*JAM2*), and phenylethanolamine-N-methyltransferase (*PNMT*). 

Pathway enrichment analysis revealed that the identified DEGs were significantly associated with two pathways, namely proteoglycans in cancer and Epstein–Barr virus infection, which are not directly associated with oocyte quality or early embryonic development.

### 2.5. Normally vs. Unequally Cleaved Embryos

Comparison between normally and unequally cleaved embryos (Figure 2C) revealed 632 DEGs (Table S3), of which 628 were upregulated in the unequally cleaved embryos. As summarized in Table 2, DEGs have been significantly enriched in 18 pathways, 7 of which are directly related to oocyte quality and/or early embryonic development. Among these is the oxidative phosphorylation (OXPHOS) pathway, in which 37 of the 141 listed genes were found to be differentially expressed (Figure S1 and Table S4). All of these genes were upregulated in the unequally cleaved embryos and encoded by the nuclear DNA (nDNA), and none was encoded by mtDNA.

Given the large difference in DEG numbers among abnormal cleavage patterns (16, 24, and 632 DEGs for directly, reverse-, and unequally cleaved embryos, respectively), a genomic inflation factor was calculated for the unequally vs. normally cleaved embryos (see Section 4.4.1). The findings revealed a λ = 0.95, indicating no evidence of inflation of the test statistics.

### 2.6. Overlapping DEGs Between Cleavage Patterns

As summarized in Figure 2D, five DEGs overlapped across all three abnormal cleavage phenotypes, namely, *JAM2*, *ISG15*, *MAGEH1*, *IFITM1*, and *PNMT*. Eleven DEGs overlapped between directly and unequally cleaved embryos, and seven DEGs uniquely overlapped between reverse and unequally cleaved embryos (Table S5).

### 2.7. Transcriptome Comparisons Between Abnormally Cleaved Embryos

Next, we directly assessed the transcription differences between the abnormal subgroups. A comparison of directly vs. unequally cleaved embryos and of directly vs. reverse-cleaved embryos revealed only two genes that were differentially expressed: *NDUFA12* and *LOC101906178*. Both genes were upregulated in the directly and reverse-cleaved embryos relative to the unequally cleaved embryos. Comparison of unequally vs. reverse-cleaved embryos resulted in 60 DEGs, all of which were upregulated in the unequally cleaved embryos. Although not significant, notable enrichment was found for cleavage-associated pathways such as cytoskeletal regulation by Rho GTPase, cadherin signaling, and integrin signaling, which might be involved in reverse cleavage pattern (Figure S2).

## 3. Discussion

Early embryonic loss has been suggested as a factor contributing to reduced fertility in lactating cows, as most embryonic losses occur between fertilization and maternal pregnancy recognition, especially within the first 7 days post-fertilization [3,9]. Nevertheless, the mechanisms that underlie early embryonic loss remain unclear. The findings of the current study support the notion that the morphokinetics of embryo cleavage is associated with developmental competence [11,12,13,14,15,16,17,18]. In particular, abnormally cleaved embryos were less likely to develop to the blastocyst stage than normally cleaved ones. Moreover, we report here for the first time that bovine embryos that display abnormal first cleavage differ in their transcriptomic profiles from normally cleaved embryos, regardless of the specific abnormal cleavage pattern (i.e., direct, reverse, or unequal). Our findings also suggest that alterations in the transcript profile underlie abnormal embryonic divisions, which, in turn, result in developmental failure and, presumably, early embryonic loss. Interestingly, among the abnormal divisions, the unequally cleaved embryos displayed the most prominent differences in gene expression compared to the normally cleaved ones. Moreover, the gene-expression profiles examined here were mainly controlled by the maternal transcriptome because in bovine, embryonic genome activation occurs at the 8- to 16-cell stage [22,23], accompanied by progressive degradation of maternally derived transcripts and a gradual shift toward embryonic genomic control [23,28]. Accordingly, we focused on enriched pathways and highlighted genes that are directly associated with oocyte competence as well as early embryonic development.

Abnormally cleaved embryos displayed low developmental competence relative to the normally cleaved ones. However, among the three abnormal patterns, embryos with direct division patterns had the highest likelihood of developing to the blastocyst stage. Whereas some studies have also reported that directly cleaved embryos have lower developmental competence [18,21], others have claimed that the developmental potential of these embryos is similar to that of normally cleaved ones [29,30]. Moreover, blastocysts that developed from directly cleaved embryos were morphologically similar to their normally cleaved counterparts [18]. On the other hand, previous studies have demonstrated that differences in first-division patterns are associated with metabolomic profiles [20], lower hatchability [19], and lower implantation rate [31] in the blastocyst stage.

An altered transcriptomic profile detected at the blastocyst stage has been suggested to underlie abnormal divisions [18]. Here, we found 16 DEGs between directly and normally cleaved embryos, which might underlie the direct pattern. The DEGs were mainly associated with metabolic processes, as indicated by the enrichment of pantothenate and coenzyme A (CoA) biosynthesis and metabolic pathways. The pantothenate pathway involves vitamin B5, a key precursor for the biosynthesis of CoA, which is an essential cofactor in key metabolic pathways, such as the synthesis of phospholipids, protein acetylation, synthesis and degradation of fatty acids, and tricarboxylic acid cycle function [32]. Adding pantothenate to the culture media of hamster embryos increased blastocyst development from the 1-cell stage [33]. Impairment of metabolic processes, such as mitochondrial dysfunction, during oocyte maturation can lead to impairment in spindle assembly [34,35], which in turn might promote direct cleavage [36]. On the other hand, a study conducted with rhesus macaques reported that intracytoplasmic injection with oxidatively stressed sperm results in a higher proportion of embryos that cleave directly to three or more blastomeres at first cleavage [37]. Although that study suggested a paternal effect on the first-cleavage pattern, it might also accompany metabolic impairment, which could affect the pattern of the first division.

The genes *RGS4* and *INSL3* were upregulated in the directly cleaved embryos. RGS4 regulates G-protein signaling [38,39], whereas INSL3 starts meiotic progression by activating the leucine-rich-repeat-containing G-protein-coupled receptor (LGR8) [40]. Thus, alterations in the G-protein signaling pathway might underlie direct cleavage and the reduced developmental competence of these embryos.

In addition, *CYP11A1* and *HSD3B1* were highly expressed in directly vs. normally cleaved embryos. Both genes are associated with ovarian steroidogenesis, a crucial pathway for the development of follicles, oocyte maturation, and pregnancy that directly affects fertilization, embryo quality, and development [41]. Thus, the high expression of these genes is suggested to be involved in the reduced development of directly cleaved embryos. Supporting this, higher expression of these genes in bovine cumulus cells was associated with lower rates of cumulus oocyte complex (COC) development to the blastocyst stage [42]. Furthermore, upregulation of *CYP11A1* in a trophoblast cell line induced mitochondrial oxidative stress as well as increased reactive oxygen species (ROS) production and DNA damage [43].

In the current study, the occurrence of reverse cleavage was very low, and none of the formed embryos developed to the blastocyst stage. Similarly, previous studies in bovine [18,19,20] and porcine [44] embryos have reported developmental failure in reverse-cleaved embryos. Magata et al. [19] reported that reverse-cleaved embryos develop more slowly and exhibit less hatchability and a higher proportion of chromosomal aneuploidy. In contrast, a recent clinical retrospective cohort study performed on 23,813 human embryos showed that reverse-cleaved embryos that reach the blastocyst stage have the same reproductive potential as normally cleaved embryos [45]. Our comparison between normally and reverse-cleaved embryos revealed 24 DEGs. These genes were associated with biological processes related to cellular and intracellular oxygen metabolism, which are highly important throughout embryonic development.

In particular, lower expression of oxidized low-density lipoprotein receptor 1 (*OLR1*) was noted in reverse-cleaved embryos. Such alterations might subsequently lead to oxidative stress. In support of this, downregulation of *OLR1* was reported to alleviate oxidative stress in brain tissues of rats after intracerebral hemorrhage [46]. Moreover, lower expression of this gene has been associated with litter size in sheep [47] and unexplained recurrent miscarriage in women [48]. In addition, reduced expression of the interferon (IFN) alpha-inducible protein 27 (*IFI27*) gene was noted in reverse-cleaved embryos. *IFI27* downregulation has been shown to affect the mitochondrial electron chain reaction in mouse brown adipocytes [49]. Previous studies in rabbits, ovine, bovine, and humans have demonstrated the deleterious effects of hyperoxia on oocytes and embryos [50,51,52].

Oxygen levels are particularly important during the zygote-to-blastocyst transition, where essential cellular proliferation and metabolic regulation depend on sufficient oxygen supply [53,54,55]. Thus, alterations in the expression of genes in the cellular hypoxia-response pathway could underlie reverse cleavage and reduced embryonic development, but other mechanisms are also possible. For example, previous studies have suggested that the occurrence of reverse cleavage is linked to the quality of the sperm, the oocyte, or both [56,57]. Other studies have suggested that a deficient microtubule network can lead to failure in the cleavage mechanism [58,59]. Whatever the cause, the low incidence of reverse cleavage likely implies that this pattern has less impact on early embryonic loss.

The enrichment analysis revealed two pathways, namely, proteoglycans in cancer and Epstein-Barr virus infection. While these pathways are not directly associated with oocyte quality or early embryonic development, they likely reflect enrichment of fundamental biological processes rather than disease-specific mechanisms. For example, according to the Kyoto Encyclopedia of Genes and Genomes (KEGG) database, the Epstein-Barr virus infection pathway is multifaceted, involving cell cycle, DNA repair, p53 signaling, and apoptosis. Our analysis revealed three genes from this pathway: *ISG15*, *FAS* (tumor necrosis factor receptor superfamily member 6 isoform 1 precursor), and *VIM* (vimentin). FAS is involved in the p53-signaling pathway and apoptosis. DNA damage facilitates activation of the p53-signaling pathway, which activates FAS [60]. Accordingly, it can be speculated that downregulation of FAS can lead to a relatively weak DNA-repair mechanism, resulting in reverse cleavage. Supporting this, in mice, failed repair of DNA damage in the zygote disrupts cell-cycle progression during G2 arrest, leading to cytokinesis failure [61]. Early DNA damage also impairs preimplantation development and cleavage, resulting in abnormalities such as micronucleus formation and aberrant first division in mouse embryos [62].

Among the three patterns of abnormal division, the unequally cleaved embryos displayed the most profound transcriptomic changes compared to normal embryos (632 DEGs). Looking at the enriched pathways, 37 out of the 141 genes in the oxidative phosphorylation pathway were differentially expressed in these embryos (up to a 6-fold change). These DEGs operate in the subunits of the oxidative phosphorylation mitochondrial complexes, which are responsible for cellular energy production, especially during early embryogenesis [63,64]. Some reports indicate that overexpression of genes that encode the subunits of these complexes affects their stability, leading to mitochondrial dysfunction [65,66,67]. Similarly, overexpression of DEGs related to the oxidative phosphorylation pathway has also been recorded in late-cleaved porcine embryo [68].

With respect to mitochondrial complex I—NADH:ubiquinone oxidoreductase, 19 out of the 45 genes encoding this complex [69] were upregulated in the unequally cleaved embryos. Complex I is the entry point for electron transport via oxidative phosphorylation [69,70]. Moreover, a study conducted in humans reported that in healthy oocytes, complex I is suppressed to avoid ROS production, whereas the rest of the oxidative phosphorylation system remains active [71]. This conserved strategy has also been described in bovine oocytes [72]. Therefore, we cautiously speculate that the overexpression in complex I observed here is associated with impairment of the protective mechanism, resulting in the unequal cleavage phenotype.

Of the 11 genes encoding the subunits of the cytochrome c reductase complex [73], *UQCR11*, *UQCRFS1*, and *UQCRQ* were upregulated in the unequally cleaved embryos. Higher expression of *UQCR11* has been reported to attenuate apoptosis in H9c2 cardiac cells [74]. On the other hand, high expression of *UQCRFS1* is suggested to contribute to the development or progression of gastric cancer [75]. Moreover, any alteration in the expression of these subunits might lead to aberrant function of the complex, which in turn can lead to increased ROS, changes in cytochrome c release, and apoptosis [76].

With respect to cytochrome c oxidase, 7 of the 13 genes comprising this complex (*COX5B*, *COX4I1*, *COX17*, *COX7A2*, *COX6A1*, *COX6B1*, and *COX7C*) were upregulated. Higher expression of some of these genes has been associated with apoptosis in several models [77,78]. In addition, upregulation of some of these genes, in particular *COX6B1* [79], *COX7A2* [80], and *COX17* [81], has been associated with enhanced or improved mitochondrial function. Less is known regarding their higher expression in the preimplantation embryo.

With respect to ATP synthase (complex IV), higher expression of five genes (*ATPMF*, *ATP5ME*, *ATP5PD*, *ATP5F1E*, and *ATP5MC2*) was noted. ATP synthase is composed of two major subunits—F(0) and F(1), and balanced expression of the encoding genes is critical [82]. *ATP5PD*, *ATP5MC2*, and *ATP5ME* encode F(0), whereas *ATPMF* and *ATP5F1E* encode F(1), the catalytic headpiece [83]. Higher expression of *ATP5MF* and *ATP5PD* has been reported to be associated with better developmental competence in bovine blastocysts [84]. However, less is known about the higher expression of these genes at the 2-cell stage. A study conducted in porcine embryos reported that in unequally cleaved 2-cell-stage embryos, the smaller blastomeres have fewer active mitochondria than the larger ones [85], implying differential mitochondrial distribution and function between the two blastomeres. Nevertheless, further investigation is required to determine whether alterations in the expression of complex IV genes, as reported here, are also associated with unequal division.

In addition to their shared outcome of lower developmental competence, five DEGs were found in all three patterns of abnormally cleaved embryos: melanoma-associated antigen H1 (*MAGEH1*), IFN-induced transmembrane protein 1 (*IFITM1*), IFN-stimulated gene 15 (*ISG15*), junctional adhesion molecule 2 (*JAM2*), and phenylethanolamine-N-methyltransferase (*PNMT*). Interestingly, all of these genes were upregulated in the abnormally vs. normally cleaved embryos; thus, it is proposed that higher expression of each of these genes might interfere with proper embryonic development.

*MAGEH1* codes for the MAGEH1 protein that belongs to the MAGE family, which is involved in important processes such as apoptosis, germ-cell differentiation, and embryonic development [86,87]. MAGEH1 has been identified in reproductive tissues [87,88], and the regulatory elements in *MAGE* gene promoters, especially zinc finger transcription factors, are key elements for embryonic development in cattle [86].

Studies have found that IFITM1 affects early embryo development [89,90,91]. During embryonic development, IFITM1 seems to mediate repulsion of primordial germ cells from certain tissues, suggesting a context-dependent role in cell migration and guidance [92]. *IFITM1* encodes the IFITM1 transmembrane protein, which plays a role in preventing viral membrane fusion, probably by altering the lipid bilayer composition and membrane fluidity [93,94,95]. Thus, the higher expression found in the abnormally cleaved embryos might be associated with alterations in blastomere membrane composition, thereby interfering with the process of cytokinesis, leading to abnormal patterns. This assumption may be more directly relevant in the case of reverse-cleaved embryos.

The gene *ISG15* is activated in the uterus by conceptus-derived type I or II (interferons) IFNs [96,97]. Expressed in embryos from day 0 to day 8, this gene is considered an important factor in determining uterine receptivity in ruminants [98,99,100,101,102]. *ISG15* knockdown resulted in a reduced proportion of hatching blastocysts [102], implying its importance for early embryonic development, as well as in mitochondrial dysfunction [103]. On the other hand, *ISG15* and *IFITM1* are both IFN-stimulated genes, and both are associated with embryo recognition and uterine receptivity. Thus, upregulation of these genes, as reported in the current study, might subsequently alter these processes. Another important role of ISG15 is in maintaining genome stability, mainly during DNA replication [104]. Because DNA replication occurs just before the 2-cell stage [105], misexpression of the *ISG15* might have a direct impact on the first-division outcome.

The gene *JAM2*, which belongs to the junctional adhesion molecule (JAM) family, is highly expressed in the luminal epithelium of the receptive uterus in mice. This expression was found through both the morula and blastocyst stages, suggesting a role in blastocyst interaction with the uterus [106]. Both *JAM2* and *IFITM1* code for protein membranes. Whereas IFITM1 is involved in membrane composition, JAM2 seals cell-to-cell contact through the formation of tight junctions [107,108,109]. Thus, any alteration in the expression of these genes, such as the upregulation reported here, might lead to abnormal division and reduced developmental competence.

The gene *PNMT* is one of the two enzymes that are involved in catecholamine biosynthesis [110]. This gene is primarily expressed in the chromaffin cells in the adrenal medulla. Various stress conditions are known to induce *PNMT* expression, including cold stress [110] and intermittent hypoxia [111]. In mice, the earliest reported embryonic stage that expresses *PNMT* is the 8.5-day embryo [112]. Accordingly, it is suggested that the higher expression of *PNMT* observed in the abnormally cleaved embryos may reflect an association between abnormal genotype and abnormal phenotype.

To date, no direct association between metabolic pathways (such as mitochondrial activity, ATP levels, or ROS production) and abnormal cleavages has been reported. Our transcriptomic data indicate that unequally cleaved embryos display a relatively high number of DEGs that relate to the oxidative phosphorylation pathway; moreover, the directly and unequally cleaved embryos also expressed gene enrichment in the metabolic pathway, supporting the mechanism suggested to underlie these abnormal cleavages. During oocyte maturation, proper mitochondrial activity is crucial for completion of meiosis and cytoplasmic maturation; this includes microtubule polymerization, actin cytoskeleton remodeling, chromosomal congression, and spindle orientation [113]. Moreover, embryonic development is accompanied by metabolic changes from the oocyte to the blastocyst stage [114]. It is therefore possible that mitochondrial dysfunction through oocyte maturation and/or the first embryonic division underlies abnormal cleavage patterns; this point should be further examined in a functional study.

## 4. Materials and Methods

### 4.1. Culture Media

All reagents were purchased from Merck-Sigma (Rehovot, Israel) unless otherwise specified. All culture media were prepared and are routinely used in our laboratory [18,115,116]. These include Hepes–Tyrode’s lactate (HEPES-TL) as the working medium, and the culture media: in vitro fertilization medium (IVF-TL), oocyte maturation medium (OMM), and potassium simplex optimized medium (KSOM). HEPES-TL was supplemented with 0.3% (*w*/*v*) bovine serum albumin (BSA), 0.2 mM sodium pyruvate, and 0.75 mg/mL gentamicin (HEPES-TALP). OMM contained TCM-199 with Earle’s salts supplemented with 10% (*v*/*v*) heat-inactivated fetal bovine serum (Sartorius, Goettingen, Germany), 0.2 mM sodium pyruvate, 50 μg/μL gentamicin, 1.32 μg/mL porcine Folltropin-V (Vetoquinol, Magny-Vernoi, France), and 2 μg/mL estradiol. IVF-TL was supplemented with 0.6% (*w*/*v*) essential fatty acid-free BSA, 0.2 mM sodium pyruvate, 0.05 mg/mL gentamicin, and 0.01 mg/mL heparin (IVF-TALP). Potassium simplex optimized medium (KSOM), which was used for embryo culture, contained 95 mM NaCl, 2.5 mM KCl, 0.35 mM KH_2_PO_4_, 0.2 mM MgSO_4_·7H_2_O, 0.8% (*v*/*v*) sodium lactate, 0.2 mM sodium pyruvate, 0.2 mM d(+)-glucose, 25 mM NaHCO_3_, 1 mM l-glutamine, 0.01 mM ethane-1,2-diyldinitrilo tetra-acetic acid (EDTA), and 0.01 mM phenol red supplemented with 1.7 mM CaCl_2_·2H_2_O, 0.1 mg/mL polyvinyl alcohol, 10 μL/mL essential amino acids (Thermo Fisher Scientific, Waltham, MA, USA), and 5 μL/mL non-essential amino acids (Thermo Fisher Scientific), with the addition of 100 U/mL penicillin-G and 0.1 mg/mL streptomycin.

### 4.2. In Vitro Embryo Production

In vitro production (IVP) of embryos was performed as previously described [18,115,116]. In brief, COCs of Holstein breed cows were aspirated from ovaries collected from a local slaughterhouse. This IVP model of bovine embryos was approved by the Ethics Committee of the Hebrew University of Jerusalem (AG-22-16883-1; approval Date: 4 February 2022). After aspiration, COCs were washed in HEPES-TALP and transferred for maturation in OMM medium for 22 h at 38.5 °C and 5% CO_2_. Then, putative matured COCs were fertilized using frozen-thawed sperm from the same bull (purchased from SION Ltd. Company, Hafez Haim, Israel). In vitro fertilization was conducted in IVF-TALP medium supplemented with PHE (0.5 mM penicillamine, 0.25 mM hypotaurine, and 25 µM epinephrine in 0.9% *w*/*v* NaCl) to enable in vitro sperm capacitation. COCs and sperm were co-incubated for 18 h at 38.5 °C and 5% CO_2_. Putative zygotes were then denuded of their cumulus cell by a ~30 s vortex in HEPES-TALP and individually transferred into drops of 25 μL of KSOM covered with mineral oil into a pre-equilibrated CultureCoin dish (Esco Medical Technologies, Kaunas, Lithuania). The dish was placed in an incubator equipped with a time-lapse system (Miri TL; Esco Medical Technologies, Ramučiai, Lithuania). Culture conditions in all chambers were set to 38.5 °C, 5% CO_2_, 90% N_2_, and 5% O_2_. All videos were analyzed with Miri TL Viewer software (version 7.0) and assessed by a single trained observer with a random double check. Cleavage-pattern classification was predefined based on standardized criteria, as done previously [18,116].

#### Morphokinetic Analysis

Each embryo was individually evaluated for several morphokinetic parameters, such as the pattern of first cleavage and the time of division through embryonic development. The time of fertilization, i.e., the initiation of oocyte incubation with spermatozoa, was defined as time = 0. Note that in the IVF procedure, the actual time of fertilization is unknown, and it might differ between individual oocytes; therefore, images of each embryo were acquired every 5 min to minimize variability associated with fertilization timing. First cleavage patterns were defined as previously reported by our laboratory [18]. In brief, normal cleavage was defined as the zygote division into two equal blastomeres. Abnormal cleavage was defined as follows: direct cleavage, in which the zygote divides directly into three blastomeres; reverse cleavage, in which the zygote divides into two blastomeres that are then merged again into one cell; or unequal cleavage, where the zygote divides into two unequally sized blastomeres.

### 4.3. Collection of First-Cleaved Embryos

Each classified embryo (i.e., directly, unequally, or reverse-cleaved) was washed several times in phosphate-buffered saline (PBS), with the addition of 1 mg/mL polyvinylpyrrolidone (PVP) at pH 7.4. Then, each embryo was loaded into a 1.7 mL DNA/RNA-free tube, snap-frozen in liquid nitrogen, and stored at −80 °C for further analysis. Embryos whose records were unclear or partially filmed were excluded from the analysis.

### 4.4. RNA Sequencing Analysis

RNA sequencing was performed by the Genomic Applications Lab, Faculty of Medicine, at the Hebrew University, Israel. RNA-seq libraries were prepared using the SMART-Seq^®^ mRNA LP (with UMIs) kit (TAKARA Bio 63472; London, UK), following the manufacturer’s protocol, with the primary modification of using cells as the starting material. Libraries were eluted in 25 µL of elution buffer (Qiagen; Hilden, Germany). Library quality was assessed using the Agilent TapeStation 4200 (Agilent; Santa Clara, CA, USA), and concentrations were measured with the Qubit 4 (Thermo Fisher Scientific; Waltham, MA, USA). Sequencing was performed on the Illumina NovaSeq 6000 (Illumina, San Diego, CA, USA), as a single-read run of 122 bp, as previously described [117].

#### 4.4.1. RNA-Seq Processing and Quantification

Raw single-end bovine RNA-seq reads were processed in R (v4.04) using the Bioconductor package Rsubread [118]. A genome index was built from the Bos taurus ARS-UCD1.2 reference assembly [119]. Then, reads were aligned in RNA mode to produce coordinate-sorted BAM files, as recommended for spliced alignment with Rsubread. Gene-level counts were generated with feature counts using Ensembl gene annotation (release 109), with exons as features aggregated to genes via the gene_id attribute, reverse-strand assignment, and inclusion of multi-overlap and multi-mapping reads, using the arguments: GTF.featureType=“exon”, GTF.attrType=“gene_id”, useMetaFeatures=TRUE, strandSpecific=2, allowMultiOverlap=TRUE, countMultiMappingReads=TRUE. Counts were converted to transcript per million (TPM), first by dividing each gene’s count by its length in kilobases (Reads Per Kilobase, RPK) and then by calculating a scaling factor by summing all RPK sample values and dividing by one million. Finally, TPM was computed by dividing each gene’s RPK value by the scaling factor [120].

Analysis of differential expression was performed with DESeq2 [121], using a design with condition as a single fixed factor (design = ~condition). Size-factor normalization used the median-of-ratios method, dispersion was estimated per gene with empirical Bayes shrinkage, and Wald tests were computed for pairwise contrasts among conditions, with Benjamini–Hochberg adjustment for multiple testing. Result tables were exported for each comparison. DEGs were identified using a significance threshold of *p* < 0.05 and an absolute log2 fold change ≥1.

To evaluate whether the large number of DEGs in the unequally vs. normally cleaved embryo comparison reflected systematic inflation of the statistical tests, we calculated the genomic inflation factor λ from the distribution of gene-level test statistics:

(1) $$\lambda =\frac{median(\chi_{observed}^{2})}{0.4549}$$

A λ value close to 1 is interpreted as indicating no substantial inflation.

#### 4.4.2. Bioinformatics Functional Analysis

A heatmap visualization was generated to represent the relative expression levels and hierarchical clustering between samples. PCA was applied to the normalized expression data (i.e., TPM) to assess global variance and identification of clustering patterns between groups. Both heatmap and PCA were generated by the Integrated Differential Expression and Pathway analysis website (iDEP v2.4.4).

Enrichment analysis of gene function and DEG clustering was conducted using the ShinyGO (version 0.82) by employing the default Ensembl Bos taurus GO annotation as background. Enrichment was considered statistically significant at adjusted *p*  ≤  0.05 after accounting for FDR according to the Benjamini–Hochberg correction for multiple testing. Pathway-enrichment analysis was conducted using ShinyGO, which retrieves KEGG annotations from the KEGG database. DEGs identified in the three pairwise comparisons were analyzed for overlaps using the Venny 2.1 software.

### 4.5. Statistical Analysis

Data were analyzed using JMP Pro-15 software (SAS Institute, Inc., 2004, Cary, NC, USA). Cleavage pattern, cleavage rate, and blastocyst formation rate were analyzed as proportional/categorical data using the Chi-square test followed by Fisher’s exact test.

Embryo kinetics, i.e., time of first cleavage, was analyzed using the Kruskal–Wallis test, followed by the Wilcoxon test for pairwise comparisons.

Gene-expression levels, quantified as log2(TPM)-transformed, were analyzed using one-way ANOVA to assess differences between experimental groups. To identify which pairs of groups differed significantly, Tukey’s honestly significant difference (HSD) post-hoc comparison tests were performed.

### 4.6. Experimental Design

The study included two sets of experiments. For all experiments, IVP was performed, for which COCs were aspirated, in vitro matured, and fertilized. Thereafter, the putative zygotes were individually cultured in an incubator equipped with a time-lapse system. The experiments were conducted during the winter season of 2023–2024 to avoid any confounding effects on the oocytes due to the hot season [122,123].

The first set of experiments aimed to associate the first cleavage pattern with the potential of the cleaved embryo to develop to the blastocyst stage. For that, putative zygotes were in vitro cultured for ~190 h to allow development to the blastocyst stage. The first cleavage patterns were defined retrospectively for each embryo as normal or abnormal, and embryonic development was further documented. The first experiment included 15 IVP runs with 294 COCs, 257 cleaved embryos, and 55 blastocysts.

The second set of experiments aimed to identify transcriptomic changes associated with an abnormal first cleavage pattern. For that, embryos were collected immediately after the first cleavage occurred, within 26–32 hpf, and were characterized according to their morphokinetic pattern as normally cleaved (*n* = 6), directly cleaved (*n* = 6), unequally cleaved (*n* = 6), or reverse-cleaved embryos (*n* = 5). Thereafter, embryos were snap frozen and subjected to RNA extraction and RNA-seq analysis.

## 5. Conclusions

This study provides comprehensive transcriptomic profiles of first-cleavage bovine embryos, focusing on differences between normal and abnormal patterns of the first division. Our data reveal that while each group of abnormally cleaved embryos (i.e., direct, unequal, and reverse cleavage) exhibited a unique set of DEGs, five of the DEGs were shared by all three. These overlapping genes may therefore serve as potential markers associated with impaired cleavage. The findings suggest an association between aberrations in the maternal transcriptome, particularly in transcripts involved in fundamental signaling pathways, and abnormal cleavage. This may be related to the mechanism underlying very early embryonic loss, potentially involving impaired regulation of the maternal mRNA degradation. Overall, this study provides new insights into the molecular features associated with abnormal division and expands our understanding of reproductive biology and embryogenesis. However, further studies are required to clarify several open questions raised by this work.

## Figures and Tables

**Table 1:** Association between morphokinetic patterns and embryonic development.

Morphokinetic Pattern	Cleaved Embryos/Total COCs (%)	Cleaved Embryos/Total Cleaved (%)	Blastocyst/Total Cleaved Embryos (%)	Time of First Cleavage (hpf)
Normal	134/294 (45.1%) ^a^	134/257 (52.1%) ^a^	53/134 (39.6%) ^a^	30.08 ± 0.3 ^a^
Direct	76/294 (25.8%) ^b^	76/257 (29.6%) ^b^	8/76 (10.5%) ^b^	34.39 ± 0.81 ^b^
Reverse	2/294 (0.7%) ^b^	2/257 (0.8%) ^b^	0/2	30.63 ± 0.89 ^ab^
Unequal	45/294 (15.3%) ^b^	45/257 (14.0%) ^b^	3/45 (6.7%) ^b^	35.1 ± 1.15 ^b^

Cleavage and blastocyst formation rates were analyzed using the chi-square test followed by Fisher’s exact test. Time of first cleavage was analyzed using the Kruskal–Wallis test, followed by the Wilcoxon test for pairwise comparisons. ^a,b^ Different superscript letters indicate statistically significant differences from the normally cleaved embryos within each column (*p* < 0.05). COCs, cumulus oocyte complexes; hpf, hours post-fertilization.

**Table 2:** Gene ontology enrichment analysis of DEGs between normally and abnormally cleaved embryos.

Morphokinetic Pattern	Pathway	Genes in the Pathway (n)	Gene Count	FDR
Direct	Pantothenate and CoA biosynthesis	20	1	0.05
	Ovarian steroidogenesis	60	2	0.01
	Steroid hormone biosynthesis	73	2	0.01
	Metabolic pathways	1539	5	0.02
Unequal	Ribosome	143	61	<0.0001
	Oxidative phosphorylation	141	37	<0.0001
	Thermogenesis	234	47	<0.0001
	Proteosome	46	8	0.0004
	Retrograde endocannabinoid signaling	148	20	<0.0001
	HIF-1 signaling pathway	105	9	0.03
	Metabolic pathways	1528	84	<0.0001

## Data Availability

The original contributions presented in this study are included in the article/Supplementary Materials. Further inquiries can be directed to the corresponding author.

## Supplementary Materials

The following supporting information can be downloaded at: https://www.mdpi.com/article/10.3390/ijms27114885/s1.
