# Supplementary material for: Developmental Morphokinetics and the Transcriptomic Profile of Bovine First-Cleaved Embryos: Normal vs. Abnormal Divisions
Source: Int J Mol Sci. 2026 May 28;27(11):4885. doi: 10.3390/ijms27114885 (PMC13256426; doi:10.3390/ijms27114885)
Supplement: Supplementary file 1 [file ijms-27-04885-s001.zip › Supplementary Material.pdf]

Supplementary Material for: Developmental Morphokinetics and the Transcriptomic Profile of Bovine First-Cleaved Embryos: Normal vs. Abnormal Divisions

Ariel Michaelov [1,†], Dorit Kalo [1,†], Moran Gershoni [2] and Zvi Roth [1,*]

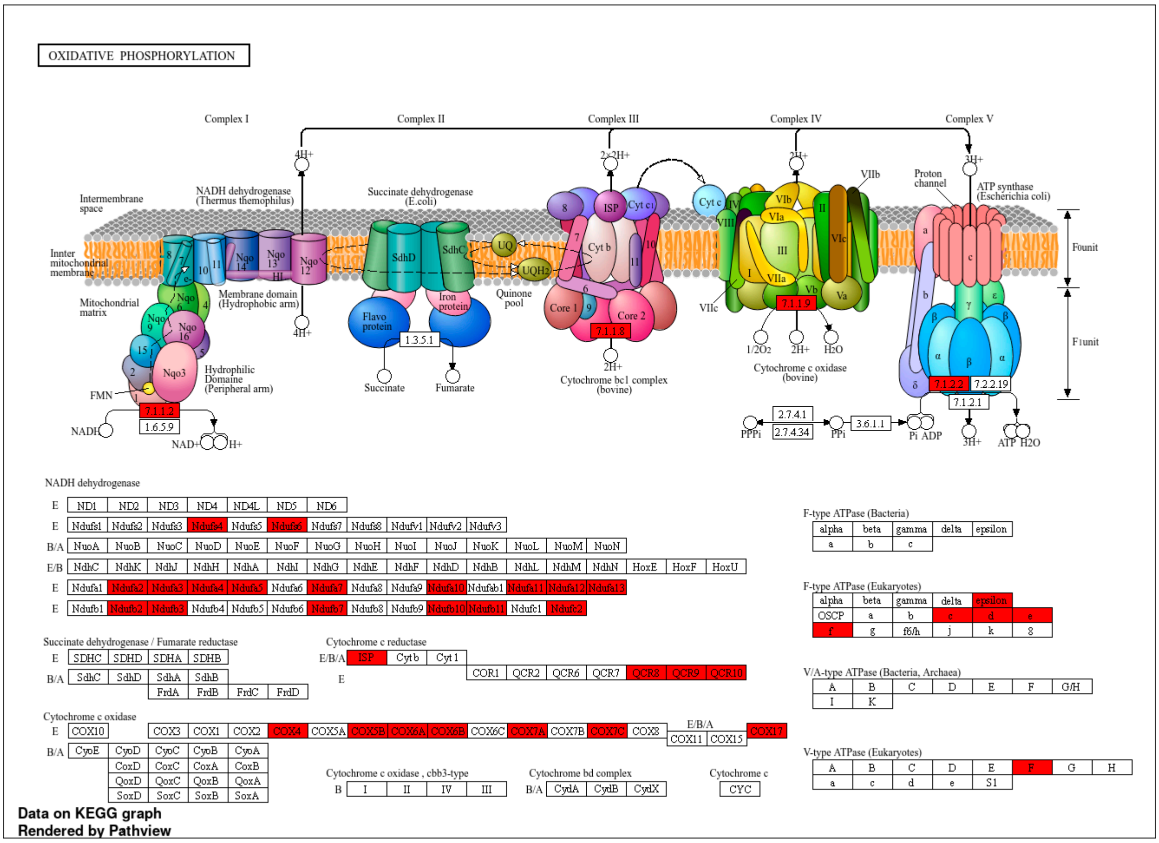

Figure S1. Oxidative phosphorylation pathway (adopted from KEGG). KEGG analysis illustration of the oxidative phosphorylation complexes with the DEGs found between the unequally- vs. the normally-cleaved embryos marked in red (this figure is complementary to Table 3).

Table S4. List of the DEGs involved with oxidative phosphorylation pathway.

Gene name	Oxidative phosphorylation complex	*Log2Fold Change	Fisher exact test (P value)
NDUFB11	NADH:ubiquinone oxidoreductase	5.1	0.0002
NDUFB10		4.5	0.0001
NDUFC2		4.4	0.006
NDUFB3		3.4	<0.0001
NDUFA3		3.34	0.0007
NDUFA5		3.3	0.001
NDUFA2		3	0.006
NDUFA11		2.6	0.0009
NDUFB11		5.1	0.0002
NDUFS4		2.6	0.01
NDUFS6		3.7	0.0009
NDUFA4L2		2.5	0.005
NDUFA12		2.4	0.001
NDUFA13		2.35	0.004
NDUFA7		2.32	0.006
NDUFB7		3.1	0.006
NDUFA10		2.18	0.004
NDUFB2		2.14	0.003
NDUFA1		4.67	0.02
UQCR11	Cytochrome c reductase	6.11	0.0001
UQCRFS1		3.7	0.0008
UQCRQ		2.8	0.0002
COX5B	Cytochrome c oxidase	4	0.0001
COX4I1		2.9	0.001
COX17		2.8	0.006
COX7A2		2.45	0.002
COX6A1		3.5	0.0009
COX6B1		3.18	0.007
COX7C		3.61	0.03
ATP5MF	ATP synthase	3.6	0.002
ATP5ME		3.2	0.002
ATP5PD		3	0.007
ATP5F1E		2.9	0.1
ATP5MC2		2.45	0.001

* Log2 Fold Change in the expression of the genes in the unequally-cleaved embryos relative to the normally- cleaved embryos.

Table S5. List of the overlapping DEGSs between the normally- vs. abnormally-cleaved embryos.

**Overlapping DEGs**	**Ensembl ID**	**Gene name**
Unequally and directly-cleaved embryos	ENSBTAG00000000603	JAM2
	ENSBTAG00000005077	CXCL12
	ENSBTAG00000006676	FIBIN
	ENSBTAG00000006769	HSD3B1
	ENSBTAG00000006934	CYP11A1
	ENSBTAG00000009938	XCL1
	ENSBTAG00000011139	BCHE
	ENSBTAG00000011494	PYGL
	ENSBTAG00000011731	PNMT
	ENSBTAG00000014707	ISG15
	ENSBTAG00000016357	VNN2
	ENSBTAG00000025775	INSL3
	ENSBTAG00000039090	MAGEH1
	ENSBTAG00000046277	RGS4
	ENSBTAG00000047635	MRO
	ENSBTAG00000048470	IFITM1
Unequally and reversed-cleaved embryos	ENSBTAG00000000603	JAM2
	ENSBTAG00000011731	PNMT
	ENSBTAG00000014707	ISG15
	ENSBTAG00000039090	MAGEH1
	ENSBTAG00000048470	IFITM1
	ENSBTAG00000003152	IFI27
	ENSBTAG00000007239	TNFAIP6
	ENSBTAG00000008184	CCT6A
	ENSBTAG00000012623	NDP
	ENSBTAG00000016800	SEMA6D
	ENSBTAG00000018463	VIM
	ENSBTAG00000019018	LOC112441484
Directly- and reversed-cleaved embryos	ENSBTAG00000000603	JAM2
	ENSBTAG00000011731	PNMT
	ENSBTAG00000014707	ISG15
	ENSBTAG00000039090	MAGEH1
	ENSBTAG00000048470	IFITM1

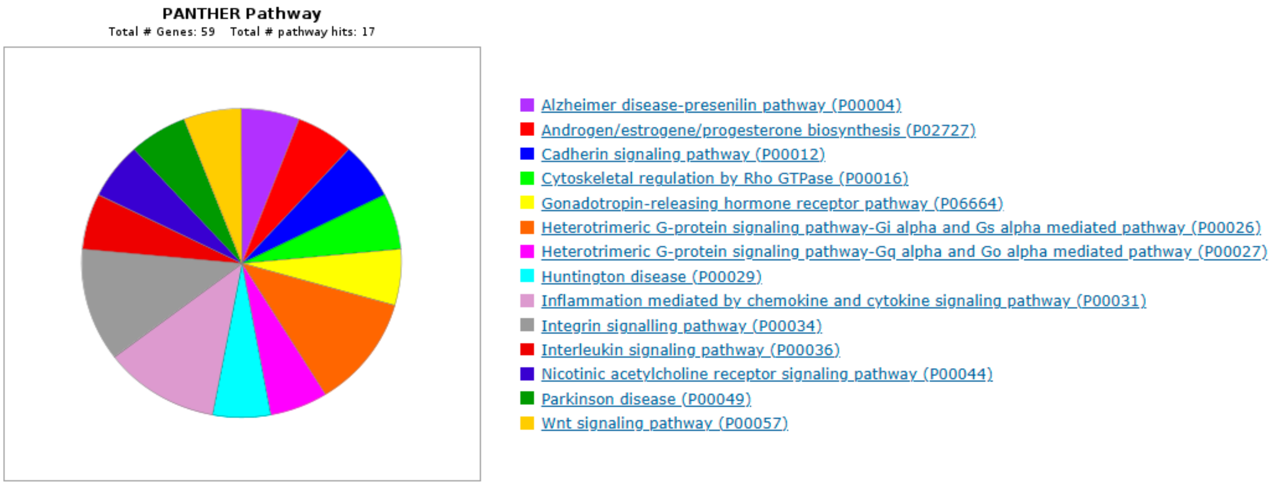

Figure S2. Pathway classification of DEGs between unequally-cleaved embryos vs. reverse-cleaved embryos using PANTHER. Among the pathway list, the cadherin signaling, cytoskeletal regulation by Rho GPTase and integrin signaling pathways are suggested to be associate with abnormal cleavage, mainly the reverse pattern.
